# Modulation of the Catalytic Properties of Lipase B from *Candida antarctica* by Immobilization on Tailor-Made Magnetic Iron Oxide Nanoparticles: The Key Role of Nanocarrier Surface Engineering

**DOI:** 10.3390/polym10060615

**Published:** 2018-06-05

**Authors:** Mario Viñambres, Marco Filice, Marzia Marciello

**Affiliations:** 1 Department of Biomaterials and Bioinspired Material, Materials Science Institute of Madrid (ICMM-CSIC), Sor Juana Inés de la Cruz 3, Cantoblanco, 28049 Madrid, Spain; mariovinambrespanizo@gmail.com; 2 Department of Chemistry in Pharmaceutical Sciences, Faculty of Pharmacy, Complutense University (UCM), Plaza Ramón y Cajal, 28040 Madrid, Spain; 3 National Research Centre for Cardiovascular Disease (CNIC), C/Melchor Fernández-Almagro 3, 28029 Madrid, Spain; 4 Biomedical Research Networking Center for Respiratory Diseases (CIBERES), C/Melchor Fernández-Almagro 3, 28029 Madrid, Spain

**Keywords:** colloid surface engineering, magnetic iron oxide nanoparticles, oriented immobilization, lipase, catalysis, nanotechnology, nanobiocatalyst, freeze-drying

## Abstract

The immobilization of biocatalysts on magnetic nanomaterial surface is a very attractive alternative to achieve enzyme nanoderivatives with highly improved properties. The combination between the careful tailoring of nanocarrier surfaces and the site-specific chemical modification of biomacromolecules is a crucial parameter to finely modulate the catalytic behavior of the biocatalyst. In this work, a useful strategy to immobilize chemically aminated lipase B from *Candida antarctica* on magnetic iron oxide nanoparticles (IONPs) by covalent multipoint attachment or hydrophobic physical adsorption upon previous tailored engineering of nanocarriers with poly-carboxylic groups (citric acid or succinic anhydride, CALB_EDA_@CA-NPs and CALB_EDA_@SA-NPs respectively) or hydrophobic layer (oleic acid, CALB_EDA_@OA-NPs) is described. After full characterization, the nanocatalysts have been assessed in the enantioselective kinetic resolution of racemic methyl mandelate. Depending on the immobilization strategy, each enzymatic nanoderivative permitted to selectively improve a specific property of the biocatalyst. In general, all the immobilization protocols permitted loading from good to high lipase amount (149 < immobilized lipase < 234 mg/g_Fe_). The hydrophobic CALB_EDA_@OA-NPs was the most active nanocatalyst, whereas the covalent CALB_EDA_@CA-NPs and CALB_EDA_@SA-NPs were revealed to be the most thermostable and also the most enantioselective ones in the kinetic resolution reaction (almost 90% ee R-enantiomer). A strategy to maintain all these properties in long-time storage (up to 1 month) by freeze-drying was also optimized. Therefore, the nanocarrier surface engineering is demonstrated to be a key-parameter in the design and preparation of lipase libraries with enhanced catalytic properties.

## 1. Introduction

In recent years, the global population growing and economy improvement in many countries has been leading to an increase of industrial life products with consequent generation of waste responsible of environmental problems and worsening of human life quality [1]. For these reasons, the quest for alternative technologies able to produce a wide set of industrial products while reducing consumption of resources and the impact on the environment is largely growing. To this end, biotechnology—or the use of bio-materials and green production processes—is a well-established reality [2,3]. Within this research field, enzymatic processes are very promising and sustainable alternatives to the conventional chemical methods [1,4]. Indeed, the enzymes are proteins able to catalyze a vast set of bio-chemical reactions. Enzymatic processes show several advantages with respect to the chemical ones in order to obtain final desired products: mild reaction conditions, simple operational procedures, high productivity, specificity, high regio- and enantioselectivity [3].

Within the different enzymes classes, lipases are the most used biocatalysts finding a wide application in different chemical reactions, including hydrolysis, acetylation, esterification in water-restricted media and kinetic resolution of racemates [5]. Their multiple applications are due to their resistance in organic solvents, thermal stability, high regio- and enantioselectivity, stability to a wide operational pH ranges, very high versatility and a broad specificity [6,7,8]. However, the practical application of enzymes in chemical processes still presents some drawbacks: their denaturation in some reaction conditions and their solubility in the reaction media makes difficult their recovery and reuse. Furthermore, many industrial synthetic processes require operating under harsh conditions in which the biocatalyst shows poor or negligible activity and stability such as, for example, high temperature, high pressure and presence of organic co-solvents [9]. 

To overcome all these limitations and to improve at the same time the overall catalyst efficiency, enzyme modifications are required and many different elegant approaches have been proposed [10,11]. One the most extensively used, is the enzyme immobilization on heterogeneous carriers [12,13,14]. By this strategy, improvement of enzyme stability (but only if multi bond attachment is obtained), activity, selectivity and/or specificity, resistance to inhibitors or chemicals, even enzyme purity may be enhanced [13,15].

With the aim of improving the stability, all the catalytic properties, the reusability and the biocatalyst application scope, various approaches immobilizing the enzyme on a heterogeneous support have been performed (e.g., physical adsorption, covalent attachment or encapsulation) [16,17,18]. Among all these strategies, covalent attachment is the mostly common used, due to the wide possibility of surface functionalization of selected carriers [19,20]. In the specific case of lipases, the most powerful approach for their immobilization and improvement of their catalytic properties is the physical adsorption on hydrophobic surfaces. In fact, when a lipase enters in contact with a hydrophobic surface, its open form is stabilized and the catalytic site became available to be reached by the substrates present in the reaction medium, finally leading to a considerable catalytic activity improvement. This phenomenon is called interfacial activation [21]. This method is lipase-specific and, in the vast majority of cases, it yields more active and selective catalysts, being especially important for advanced selective reactions [22]. However, with this immobilization protocol, in some specific cases enzyme desorption at high temperatures, or in the presence of substrates/products with detergent properties could happen.

Due to the advancements reported on in the nanotechnology field, the interest in using nanomaterials as carriers for large biomolecules immobilization has rapidly increased [23,24]. For example, thanks to their higher capture efficiency, high surface-to-volume ratio, faster reaction kinetics and minimal sample volume, nanomaterials have encountered a wide application in the preparation of advanced immunosensors [25,26]. To this end, the correct immobilization of antibodies on solid-liquid interfaces is a critical issue in the development of such systems. For example, Baniukevic and coworkers have clearly demonstrated the key relevance of antibody oriented immobilization on magnetic gold nanoparticles to develop a sensitive SERS-based sandwich immunoassay [27].

Among others, the main advantages in using this kind of support are: minimum diffusional limitation, maximum surface area per unit mass and high enzyme loading. However, it is important to keep in mind that the nanomaterials as carrier for enzyme immobilization also present some limitations for their application in biocatalysis. For example, being the enzyme immobilized on the nanocarrier surface, it could undergo to inactivation in stirred systems. This drawback can be overtaken due to the intrinsic colloidal stability of nanocarriers that permit avoiding stirring, simply using, for example, rotating inversion over a carousel. In this way, a homogeneous dispersion of nanocatalyst in the reaction mixture can be easily achieved under homogeneous and mild mixing conditions.

Among the available nanomaterials, iron oxide nanoparticles (IONPs) are very interesting nanocarriers due to their unique magnetic properties that confer them the ability to be recovered from the reaction media by magnetic attraction and to dissipate the heat generated in presence of alternating magnetic field [28,29]. This non-conventional heating strategy is known as magnetic hyperthermia and it is becoming an attractive approach for industrial applications [30]. Its mechanistic aspect on bioprocesses catalyzed by enzymes immobilized on magnetic particles have been already investigated and elucidated [30,31]. In fact, only when directly immobilized on the magnetic particle surface, an enzyme can be selectively activated by the heat generated from the particles and then its catalytic properties can be tuned [30]. 

However, independently of the selected heating strategy, beside the activity improvement, also thermal stability, specificity and enantioselectivity must be properly modulated and evaluated together with recyclability and storage properties in order to develop a powerful heterogeneous biocatalyst useful for advanced bioprocesses with industrial potential. The most useful strategy to achieve this overall goal is designing enzyme immobilization protocols on properly functionalized surfaces. 

As previously outlined, the lipase immobilization on nanoparticles mainly focuses on the enzymes leaching prevention and the enhancement of their catalytic properties. To that end, typical strategies for immobilizing *Candida antarctica* lipase B onto nanoparticles are covalent bond attachment and lipase adsorption on hydrophobic surface. In the first case, the most used approaches are based on immobilization on silica nanoparticles [32] and iron oxide nanoparticles combined with silica [33] or different polymers [34,35] in a core-shell manner. In these cases, the covalent attachment of lipase B is improved by increasing the proper reactive groups on the nanoparticles surface, in order to promote covalent bond formation with lipases. 

Hence, in this work, we have synthesized magnetic IONPs by co-precipitation method. After that, we have enriched their surface with carboxylic acid groups using citric acid (CA-NP), succinic anhydride (SA-NP) and oleic acid (OA-NP). Driven by these tailor-made surfaces, we have then promoted two different immobilization protocols, the carbodiimide-mediated covalent immobilization on CA-NP and SA-NP and the hydrophobic physical adsorption on OA-NP. All the nanobiocatalysts were physically, chemically and magnetically characterized. Finally, to analyze the modulation extent of catalytic properties of each nanocatalyst and to directly relate it to each immobilization strategy, an interesting enantioselective bioprocess was studied at different temperatures. In more details, the kinetic resolution of racemic methyl mandelate producing the pure (*R*)-(−)-mandelic acid, an important chiral building block widely used in pharmacy and in the production of fine chemicals [36], was carried out.

## 2. Materials and Methods

### 2.1. Chemicals and Reagents

Iron(III) chloride (FeCl_3_, 27% *w*/*v*) was from VWR Chemicals (Fontenay-sous-Bois, France). Iron(II) chloride tetrahydrate (FeCl_2_·4H_2_O, ≥99%), ammonium hydroxide (NH_4_OH, 25% *v*/*v*), nitric acid (HNO_3,_ 65% *w*/*w*), iron(III) nitrate nonahydrate (Fe(NO_3_)_3_)·9H_2_O, ≥98%), citric acid (C_6_H_8_O_7_, ≥99.5%), succinic anhydride (C_4_H_4_O_3_, ≥99%), sodium phosphate monobasic (NaH_2_PO_4_, ≥99%), oleic acid (CH_3_(CH_2_)_7_CH=CH(CH_2_)_7_COOH, ≥99%), sucrose (C_12_H_22_O_11_, ≥99.5%), Ethylenediamine (NH_2_CH_2_CH_2_NH_2_, ≥99.5%), *N*-hydroxysuccinimide (C_4_H_5_NO_3_, ≥98%), *N*-(3-Dimethylaminopropyl)-*N*’-ethylcarbodiimide (C_8_H_17_N_3_·HCl, ≥98%), *p*-nitrophenyl butyrate (C_10_H_11_NO_4_, ≥98%), methyl dl mandelate (C_6_H_5_CH(OH)COOCH_3_, ≥97%), methyl (*R*)-(−)-mandelate (C_6_H_5_CH(OH)COOCH_3_, ≥99%), methyl (*S*)-(+)-mandelate (C_6_H_5_CH(OH)COOCH_3_, ≥99%) were from Sigma-Aldrich (Saint Louis, MO, USA). Lipase from *Candida antartica* fraction B (Lipozyme^®^ CALB) was a kind gift gratefully received from Novozymes (Bagsvaerd, Denmark). 

### 2.2. Magnetic Nanoparticles Synthesis

Magnetite NPs were obtained by following a modified Massart coprecipitation protocol [37]. Briefly, magnetite (Fe_3_O_4_) NPs were synthesized by adding 488 mL of an aqueous solution of FeCl_3_ 27% (*w*/*v*; 0.072 mol) and FeCl_2_·4H_2_O (0.054 mol) to 75 mL of NH_4_OH 25% (*v*/*v*). The iron solutions were slowly added (0.2 mL/s) onto the alkaline medium with the aim to obtain NPs with high core size [38]. After that the entire reaction suspension was heated at 90 °C for 1 h. 

After cooling at room temperature, the synthesized NPs were washed three times with distilled water and collected with the help of a permanent magnet.

*Acid treatment*. A standard protocol was used to oxidize magnetite to maghemite (γ-Fe_2_O_3_) and reduce surface NPs imperfections [39,40]. Briefly, 300 mL of HNO_3_ (2 M) were added to 500 mL of NPs suspension, and the mixture was stirred for 15 min. Then, the supernatant was removed by magnetic decantation and 75 mL of Fe(NO_3_)_3_ (1 M) and 130 mL of water were added to the particles. The mixture was heated to boiling temperature and stirred for 30 min [39,41]. The particles were then cooled to room temperature and, by magnetic decantation, the supernatant was replaced by 300 mL of HNO_3_ (2 M). The final solution was stirred for 15 min. Finally, the particles were washed three times with acetone and re-dispersed in water. A rotary evaporator was used to remove any acetone residue as well as for concentrating the sample. 

### 2.3. Magnetic Nanoparticles Surface Modification

The synthesized iron oxide NPs were superficially modified to generate the proper coating surface in order to promote the subsequent lipase immobilization. In particular, these NPs were coated with two different classes of molecules: hydrophilic and hydrophobic. As hydrophilic modifier, citric acid and succinic anhydride were used to provide carboxylic groups onto NPs surface while to obtain a hydrophobic surface, oleic acid was employed. 

*Citric acid (CA) coating*. For citric acid coating, a standard procedure was used [42,43]. 50 mg of IONPs resuspended in 25 mL of distilled water were added to 100 mL of a 0.1 M citric acid solution, adjusting the final pH to 2–3 value. Afterward, the mixture was sonicated and then heated at 80 °C for 30 min. The solution was centrifuged, resuspended in distilled water and finally dialyzed for 3 days in a tubing cellulose membrane (14 kDa, cut-off, Sigma-Aldrich).

*Succinic anhydride (SA) coating*. To provide carboxyl groups on NPs surface, succinic anhydride conjugation was used [44]. A solution of 100 mM succinic anhydride in 5 mM phosphate buffer pH 8 was prepared. 90 mg of iron oxide NPs were added to succinic anhydride solution (with final volume of 10 mL) and the pH was adjusted to 8. The suspension was mechanically stirred and the reaction was maintained at pH 8.0 for 16 h by adding NaOH 0.1 M. Once the reaction was finished, the mixture was magnetically decanted, washed once with ethanol, then with distilled water and finally dialyzed for 3 days in a tubing cellulose membrane (14 kDa, cut-off, Sigma-Aldrich).

*Oleic acid (OA) coating*. Surface modification was carried out by adding 1.6 g of oleic acid to the colloid containing 400 mg of Fe in 80 mL of water, keeping ultrasonic agitation at 70–80 °C for 1 h. After that, the sample was magnetically decanted, washed first with H_2_O then with ethanol (3×) and finally N_2_ dried and redispersed in toluene.

### 2.4. Enzyme Chemical Amination

The CALB lipase was modified with ethylenediamine (EDA) to increase the amine groups on its surface in order to promote a multipoint covalent immobilization by amide bond formation [45]. Briefly, 5 mL of crude lipase solution at 7.6 mg/mL (titred by Bradford assay) were added to 20 mL of 1 M EDA pH 4.75. Afterward, 49 mg of *N*-(3-Dimethylaminopropyl)-*N*′-ethylcarbodiimide were added to obtain a final concentration of 10 mM and the final pH value was quickly adjusted to 4.75. The reaction mixture was left under mild stirring up to 1.5 h at room temperature. To purify the aminated enzyme, centrifugal filter units Amicon Ultra-15 10 kDa molecular weight cut-off (Merck Millipore, Billerica, MA, USA) were used to wash and concentrate with 10 mM phosphate buffer pH 7.

### 2.5. Enzyme Immobilization on Magnetic Nanoparticles

Aminated CALB lipase (CALB_EDA_) was covalently conjugated both on citric acid and succinic acid-coated NPs by carbodiimide chemistry. 16 mL of an aqueous solution of hydrophilic coated NPs at 2 mg/mL were prepared, and *N*-hydroxysuccinimide and *N*-(3-Dimethylaminopropyl)-*N*′-ethylcarbodiimide were added obtaining a final concentration of 100 and 20 mM respectively. The final pH was adjusted to 5 and incubated with mild agitation for 1.5 h at room temperature. Then the sample was centrifuged at 7500 rpm for 10 min. The supernatant was discarded and 30 mL of pure CALB_EDA_ solution at 0.26 mg/mL were immediately added to the precipitate to resuspend it and the pH was quickly adjusted to 7.8. The final suspension was incubated for 16 h under mild stirring at room temperature and then centrifuged. The precipitated NPs were thoroughly washed with distilled water and resuspended in 10 mM phosphate buffer pH 7. The first supernatant was collected for the subsequent evaluation of the immobilization process efficiency. To confirm the covalent attachment of CALB_EDA_ on NPs, both CALB_EDA_@CA-NPs and CALB_EDA_@SA-NPs nanoderivatives were incubated in 2 M NaCl PBS for 1 h. After magnetic decantation and supernatant purification by ultracentrifugation with 3 KDa filter (to avoid eventual enzyme loss), the purified supernatant was analyzed by lipase activity and Bradford assay. Negative results were obtained in both cases confirming that CALB_EDA_ has been covalently attached to magnetic nanoparticles.

*Hydrophobic enzyme adsorption*. For OA-coated NPs, the immobilization of the enzyme was carried out by adsorption of CALB_EDA_ lipase onto hydrophobic NPs surface, as previously described for other lipases in hydrophobic supports [21,46]. Briefly, 30 mg of OA-NPs previously concentrated in 1 mL toluene by solvent evaporation under nitrogen flow were added to 30 mL of CALB lipase solution in 10 mM phosphate buffer pH 7 (ratio OA-NP: Aqueous solution (*v*/*v*) 1:30). The sample was vigorously stirred, sonicated to obtain a more homogeneous microemulsion and then left under mild stirring for 16 h. Subsequently, the NPs were magnetically decanted and the supernatant was collected to evaluate the efficiency of the lipase immobilization process. Finally, the NPs were washed several times with water and resuspended in 10 mM phosphate buffer pH 7. 

### 2.6. Lipase Activity Assay

The activity of soluble and immobilized lipase was carried out spectrophotometrically by measuring the increase in absorbance at 348 nm produced by the release of *p*-nitrophenol (pNP) in the hydrolysis of 0.4 mM of *p*-nitrophenyl butyrate (p-NPB) in sodium phosphate (25 mM) 25 °C (ε = 5150 M^−1^·cm^−1^) [47,48]. 20 µL of 50 mM p-NPB solution in acetonitrile were added to 2.5 mL of phosphate buffer 10 mM pH 7 and then 30 µL of either lipase solution or suspension were added. Enzymatic activity is presented as µmol of pNP produced per minute per mg of enzyme (IU) under the conditions above described [49]. 

Soluble lipase concentration was measured according to Bradford assay [50] by the colorimetric Coomassie Protein Assay Kit (Thermo Scientific, Rockford, IL, USA) with bovine serum albumin (BSA) as standard. 

### 2.7. Structural, Colloidal and Magnetic Properties Characterization

Particle size and shape were determined by transmission electron microscopy (TEM). The mean particle size and distribution were evaluated by measuring the largest internal dimension of 200 NPs. Afterward, data were fitted to a log-normal or Gaussian distribution to obtain the mean size and standard deviation (σ), which is considered to be representative of the absolute error of the measurement.

Fourier transform infrared spectra (FTIR) were recorded between 4000 and 250 cm^−1^ in a Bruker IFS 66 V-S spectrometer (Bruker Optics, Ettlingen, Germany). Samples were prepared by dilution of powder nanoparticles (2 wt %) in KBr and compressing the mixture into a pellet. Simultaneous thermogravimetric (TG) analysis were performed on a Seiko TG/DTA 320U thermobalance (Seiko Instruments Inc., Chiba, Japan). Powder samples were heated from room temperature to 900 °C at 10 °C/min under an air flow of 100 mL/min. 

Colloidal properties of the samples were studied in a Zetasizer Nano S, from Malvern Instruments (Malvern, UK), by dynamic light scattering (DLS). The hydrodynamic size of the particles in suspension was measured in intensity (Z-average) and in number. Each hydrodynamic value is the result of three different measurements at different dilutions to avoid errors coming from backscattering and using the scattering index for the solvent where the particles are dispersed, water or toluene in the case of OA-coated magnetic NPs. The zeta potential (ζ-potential) of NPs was also measured in a pH range of 2–10. Samples were diluted in KNO_3_ solution (10^−2^ M), and pH-values were adjusted by adding HNO_3_ 0.05 M and KOH 0.05 M.

The Fe concentration was measured with an inductively coupled plasma optical emission spectrometer (ICP-OES) PerkinElmer Optima 2100 DV (PerkinElmer Inc., Wellesley, MA, USA). For this purpose, samples were digested with *aqua regia* to oxidize the organic coating and to dissolve the particles. 

The magnetic characterization was carried out using a vibrating sample magnetometer MagLabVSM, Oxford Instruments (Abingdon, UK). Hysteresis loops, M(H), of powder samples were recorded at 250 K under a maximum magnetic field applied of 50 kOe. Determination of saturation magnetization, Ms, was calculated by fitting of magnetization curves to Langevin function using Originpro software (OriginPro 2016 Version, OriginLab Corporation, Northampton, MA, USA). Ms values were normalized to the magnetic mass of maghemite determining the iron concentration by ICP-OES. 

Heating capacities of NPs were measured with the system CELES MP 6 kW (Fives Celes, Lautenbach, France). A half-length of the sample was made to coincide with the maximum value of the magnetic field, that was placed in the middle of the coil (71 mm diameter, 100 mm height, 6 turns). The volume of the sample was 250 µL. The temperatures of the coils were controlled through a closed circuit of water maintained at 16 °C with a continuous water flow. The temperature of the samples was measured with an optical fiber thermometer and registered with a computer. The measurement was carried out applying an alternating magnetic field of 20 mT at frequency of 282 kHz and. Prior to turning the magnetic field on, the sample temperature was recorded for about 15 s to ensure thermal stability and to have a baseline for the calculation of the specific absorption rate (SAR). As the field was turned on, the temperature increase was measured either during 300 s or up to 50 °C for aqueous and toluene samples. The slope Δ*T*/Δ*t* was obtained by performing a linear fit of data (temperature vs. time) in the initial interval of time. As the measurements were performed in non-adiabatic conditions, the curve slopes Δ*T*/Δ*t* were fitted only in the first few seconds after turning the magnetic field on. The time range was selected such as the slope is maximum, typically during the first 30 s [51]. The SAR values were calculated as:

SAR = (C_liq_/cFe) (Δ*T*/Δ*t*),

where: C_liq_ is the specific heat capacity of water (4.185 J/(g·K)) or toluene (1.71 J/(g·K)) and cFe is the Fe weight concentration in the sample [52], normalizing to the iron mass (W/gFe). 

### 2.8. Enzymatic Kinetic Resolution of Racemic Methyl Mandelate *(**1**)*

The different immobilized nanocatalysts (CALB_EDA_@CA-NPs, CALB_EDA_@SA-NPs and CALB_EDA_@OA-NPs) at the concentration of 1 mg_lip_/mL in 10 mM phosphate buffer (500 μL), were added to 1.5 mL screw-sealed vessel containing 3 mM substrate in 10 mM phosphate buffer/acetonitrile (95/5 *v*/*v*) (1 mL) at pH 7 and 25 or 45 °C under continuous shaking. The conversion degree was analyzed by RP-HPLC using a Phenomenex Gemini^®^ 5 µm C18 110 Å, 250 × 4.6 mm LC column (Phenomenex Inc., Torrance, CA, USA). Samples (200 μL of reaction mixture magnetically decanted and diluted 4× with mobile phase) were injected and eluted at a flow rate of 1.0 mL/min using acetonitrile/MilliQ water (Merck Millipore, Billerica, MA, USA) (35:65, *v*/*v*) with pH adjusted to 2.8 with TFA as mobile phase. UV detection was performed at 230 nm. The acid has *R_t_* of 2.3 min while the ester has *R_t_* of 5 min.

The enantiomeric excess (ee) value was determined by chiral HPLC analysis of the reaction’s samples withdrawn as described in Table 2. The analysis conditions were: Phenomenex Lux Cellulose-1 (250 × 4.6 mm, 3 µm) column, flow of 1 mL/min; λ: 225; mobile phase: 10% (*v*/*v*) IPA in *n*-Hexane and temperature: 25 °C. *R_t_* of S-**1** was 9 min whereas *R_t_* of *R*-**1** was 15.5 min. Before their injection, the different aqueous reaction samples (400 µL) were extracted with AcOEt (500 µL). The upper organic layer was separated and dried with anhydrous Na_2_SO_4_. After solid removal, the remaining dry organic phase was carefully dried under N_2_ stream and the achieved solid was dissolved with 400 µL of mobile phase. Each sample was injected in triplicate. 

To perform the recycling studies, after reaching the desired final point, each reaction was centrifuged 10 min at 15 k rpm. The recovered catalyst was washed and centrifuged 3 times with 10 mM phosphate buffer/acetonitrile (95/5 *v*/*v*) (1 mL) at pH 7 and finally used with a new batch of reagents as previously described.

### 2.9. Thermal Stability Assay

Colloidal suspensions of the prepared coated NPs with and without CALB_EDA_ lipase were incubated in 10 mM sodium phosphate buffer at 45 and 55 °C pH 7.0. Aliquots of the suspension were periodically withdrawn (until 4 h). In the case of coated NPs without lipase, the suspension was precipitated and the supernatant was analyzed by TGA, in order to check the surface coating stability. In the presence of the immobilized lipase, enzyme activity was analyzed as described above. 

### 2.10. Enzyme Nanocatalyst Storage Evaluation

The coated magnetic supports with and without the immobilized lipase were stored in different conditions: colloidal suspensions and lyophilized form. Colloidal properties and lipase activity were monitored over the time for each different storage condition. To store the samples in powder form, the colloidal NPs suspensions were lyophilized in presence and absence of sucrose at 5% (*w*/*v*) [53] and then stored at 4 °C. The activity and colloidal properties of lyophilized form were analyzed after reconstitution of colloids by hydration. 

## 3. Results and Discussion

### 3.1. Magnetic Nanoparticles with Tailor-Made Surface Coating as Support for Enzyme Immobilization: Preparation and Characterization

**Figure 1:**
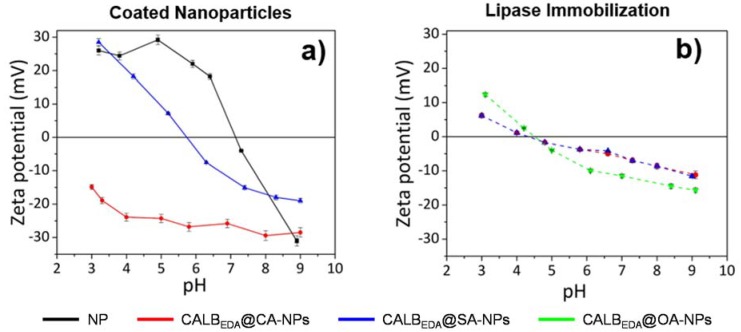
Zeta potential values vs. pH of (**a**) CA and SA coated NPs compared with uncoated NPs and of (**b**) CALB_EDA_@CA-NPs, CALB_EDA_@SA-NPs and CALB_EDA_@OA-NPs.

In this study IONPs were synthesized by co-precipitation method followed by acid treatment to increase their size distribution and crystallinity obtaining NPs with enhanced magnetic properties [39,54]. The prepared IONPs showed a core size of 13 nm (Figure S1a) and a hydrodynamic diameter around 100 nm (Table 1). Uncoated NPs resulted colloidally unstable at pH 7 because their charge was close to zero, as confirmed by ζ-potential measurements (Figure 1). The magnetization saturation (Ms) value of naked NPs was quite high in comparison with Ms of bulk maghemite (69 vs.80 emu/g, respectively) indicating that these particles can be easily recovered by a 0.1 T permanent magnet. SAR value was also measured (Table 1). This resulted in 131 W/gFe meaning that these maghemite NPs can produce a 10 Celsius degrees increase from room temperature in 90 s at 4 mgFe/mL concentration (data not shown).

**Figure 2:**
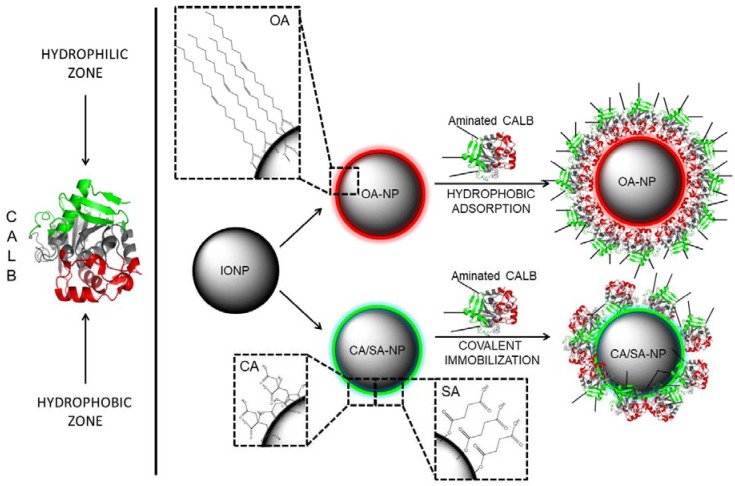
Controlled immobilization of chemically modified CALB on tailor-made magnetic iron oxide nanoparticles (IONP). (OA: oleic acid; CA: citric acid; SA: succinic anhydride).

In order to generate on NPs surface a carboxylic group layer useful for subsequent covalent enzyme immobilization, citric acid (CA) and succinic anhydride (SA) were used (Figure 2). On the other hand, to obtain a hydrophobic surface useful to promote the subsequent physical adsorption, oleic acid (OA) was employed as coating agent (Figure 2). It is expected that OA stabilizes the particles in hydrophobic organic media leading to full particle disaggregation [55].

To prove the presence of each desired coating on NPs surface, colloidal properties (hydrodynamic size and ζ-potential), FTIR and TGA of coated vs. uncoated NPs were investigated. 

The hydrodynamic sizes of the three coated-NPs were 80, 121 and 109 nm for CA-NPs, SA-NPs and OA-NPs respectively (Table 1). In the case of SA-NPs and OA-NPs, a slight increase of the aggregates size was observed after coating, whereas in the case of CA-NPs the hydrodynamic size decreased. This different behavior is probably related to a different amount of carboxylic acid per coating molecule. In fact, the three carboxyl groups of CA generate a strong electrostatic repulsion between NPs, finally resulting in the disaggregation and hydrodynamic size reduction. On the contrary, in the case of SA-coated NPs, after reaction SA generates only one carboxylic terminal group [56], being responsible for a weak electrostatic interparticle aggregation with consequent hydrodynamic size increase. The ζ-potential of the samples showed a shift of the isoelectric point from 7 to 6 for SA-NPs and to values <3 for CA-NPs (Figure 1a). These results are in agreement with the presence of higher amount of carboxylic groups on CA-NPs surface. 

**Figure 3:**
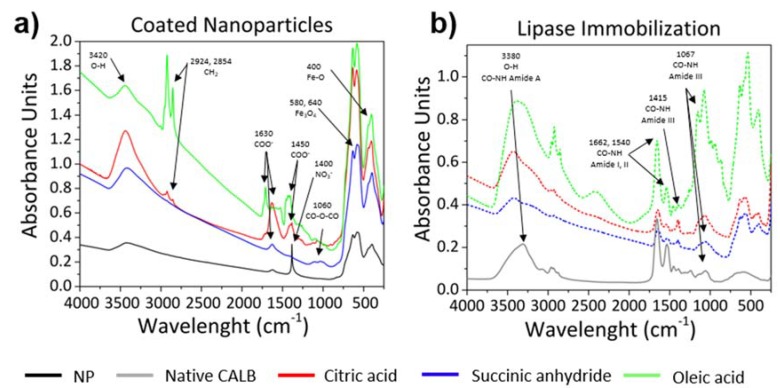
Fourier transform infrared spectra (FTIR) spectra of (**a**) CA, SA and OA coated NPs compared with the naked one and of (**b**) CALB_EDA_@CA-NPs, CALB_EDA_@SA-NPs and CALB_EDA_@OA-NPs.

FTIR spectra clearly confirmed the presence of each desired coating on the NPs surface (Figure 3a). Firstly, all samples showed the characteristic peaks of iron oxides: Fe–O vibrations at 640, 580 and 400 cm^−1^ [57]. A broad peak at 3420 cm^−1^ was also present corresponding to O–H stretching for the presence of hydroxyl group on NPs surface and carboxylic groups in the case of coated NPs. Bare NP (black line, Figure 3a) showed a significant sharp peak at 1400 cm^−1^ corresponding to N–O stretching, due to the presence of nitric acid residues after acid treatment.

OA and CA-coated NPs presented similar spectra (Figure 3a). The two peaks at 2924 and 2854 cm^−1^ can be assigned to the asymmetric and symmetric CH_2_ stretches, being much stronger in OA-coated NP as a result of its alkyl chain. The other peaks around 1630 and 1450 cm^−1^ are attributed to carboxylic group vibrations, corresponding to asymmetric and symmetric COO^−^ stretching [57]. In the case of SA-coated NP, two weak peaks were observed, one around 1630 cm^−1^, for carboxylic group, in the formation of succinic acid attached to the NP surface, and the other at 1060 cm^−1^ that can be assigned to the stretching vibration of anhydride group. Comparison of coated-NPs with the free molecules of citric acid and succinic anhydride are reported in Supplementary Materials (Figure S2). 

**Figure 4:**
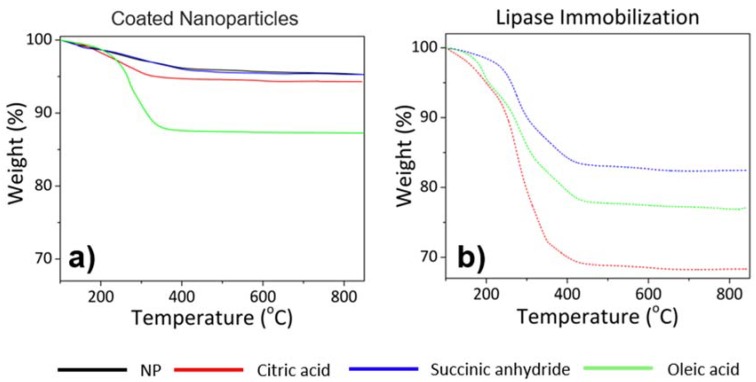
TGA curves of (**a**) CA, SA and OA coated NPs compared with the naked one and of (**b**) CALB_EDA_@CA-NPs, CALB_EDA_@SA-NPs and CALB_EDA_@OA-NPs.

The amount of the three different coatings was quantified by TGA (Figure 4a). A weight loss of ~1%, 5.7% and 12.7% was observed for SA-NPs, CA-NPs and OA-NPs respectively in comparison with bare NPs. Ms of coated NPs were also measured. The presence of coating slightly decreased Ms values, except for CA-coated NPs that showed a significant decrease (55 emu/g) (Table 1). This was probably due to the partial digestion of NPs surface after citric acid treatment, as confirmed by core size reduction (~1 nm) from TEM micrographs (Figure S1). SAR values did not vary significantly, except for the OA-coated NP that suffered a reduction up to 106.45 W/g (Table 1).

### 3.2. Controlled Enzyme Immobilization on Tailor-Made Magnetic Nanocarriers

After the full characterization, the three different magnetic nanomaterials were used as nanosized heterogeneous supports to promote the controlled immobilization of *Candida antarctica* lipase B. The aim was modulating and improving the overall catalytic properties (mainly activity, stability and selectivity) of lipase by controlled immobilization processes over nanocarriers. To that end, two immobilization strategies were studied: covalent conjugation and hydrophobic absorption on the surface of properly tailored magnetic NPs (Figure 2). In the first case, the covalent immobilization was achieved by carbodiimide-mediated formation of amide bonds between carboxylic groups present on CA- and SA-NPs surface and the amine groups of the lipase. In order to generate a strong stabilizing multipoint covalent attachment of the biocatalyst on NPs surface, previously to its immobilization reaction, the enzyme surface was aminated by chemical modification with ethylediamine (CALB_EDA_) (Figure 2) [45]. By this reaction, the carboxy group side chain residues of Asp and Glu aminoacids exposed on CALB surface have been converted into amine group through covalent reaction with EDA. Consequently, an amount of amine reactive points double than the naturally occurring ones (19 NH_2EDA_ residues vs. 9 NH_2Lys_ residues, (structural analysis carried out on PyMOL software using PDB file: 1TCA)) was created. Furthermore, this newly generated amine groups show higher reactivity than the aminoacidic side chain residues (*pKa* NH_2EDA_: 7.7 vs. *pKa* NH_2Lys_: 10.54), finally granting a strong multipoint covalent immobilization at milder reaction conditions. 

In the case of hydrophobic absorption, OA coated NPs oriented the physical adsorption of CALB_EDA_ by their hydrophobic pocket, exposing the opposite hydrophilic region toward the aqueous reaction medium (Figure 2). As a consequence of this oriented immobilization, a great change in colloidal stability of hydrophobic NPs could be promoted. In fact, by this strategy a complete switch from organic solvent to aqueous solution can been achieved [46].

After each immobilization process, the reaction yields and specific activity of the immobilized lipase was measured in order to assess the modulation extent of the catalytic properties. Consequently, any potential positive or negative variation can be directly related to each immobilization strategy. 

Hence, in terms of mass transfer, the covalent immobilization on CA-NPs was the best one allowing the immobilization of 234 µg lipase/mg_Fe_ (Table 1). On the other hand, the immobilization protocols carried out on SA-NPs and OA-NPs retrieved lower and quite similar results, being 149 and 162.7 respectively the µg of CALB_EDA_ immobilized on each mg of Fe (Table 1).

To analyze the impact of each immobilization protocol on catalytic activity, the hydrolysis of pNPB ester was assayed. The activity of free CALB_EDA_ was determined as reference obtaining a 22 IU/mg_lip_ value (Table 1). As general trend, all the three immobilization strategies led to an activity loss in comparison to the free enzyme. In more details and in agreement with similar works reported in literature [10], CALB_EDA_@OA-NPs was revealed to be the strategy that preserved the highest catalytic activity, being 13.5 IU/mg_lip_ its specific activity (Table 1). On the other hand, CALB_EDA_@CA-NPs and CALB_EDA_@SA-NPs showed lower specific activities, 5.73 and 8.28 IU/mg_lip_ respectively (Table 1).

It is interesting to remark that after colloidal and morphological characterization of CALB_EDA_ nanoderivatives, in all cases the presence of lipase increased the hydrodynamic sizes preserving the nucleus size (Table 1 and Figure S1). The colloidal stability properties were quite similar in all cases. In fact, all the three nanoderivatives resulted colloidally stable at pH 7, showing an isoelectric point value around 5 and zeta-potential profiles very similar in all the range of pHs (Figure 1b).

The presence of the enzyme on NPs surface was confirmed by FTIR and TGA (Figure 3b and Figure 4b). The IR spectra of the three immobilization types showed peaks of amide groups due to the polypeptide chain of proteins. The peak around 3420 cm^−1^ was broader than the spectra of coated-NPs (Figure 3b) due to the contribution of N-H stretching in primary amines at 3280 cm^−1^, which is characteristic of the amide A region [58,59]. The other peaks that appear at 1662, 1540, 1415 and 1067 cm^−1^ are assigned to amides I, II and III vibration region, characteristic of secondary structures of proteins, (β-sheet, unordered, α-helical and turns structures), and the symmetrical bending of the N–H bonds corresponding to the NH_3_^+^ group of the amino acids [59]. TGA analysis showed a weight loss of 24%, 12% and 11% for the lipase immobilized in CA, SA and OA-coated NPs, respectively (after eliminating the contribution of each surface coating) (Figure 4b). The weight losses here obtained are in agreement with the immobilization yields previously calculated. 

Magnetic properties were also evaluated in order to assess whether the attached lipase could negatively impact on the magnetic moment (Ms) and on the heating ability (SAR) of the NPs (Table 1). Effectively, the presence of the enzyme reduced the Ms values. This decrease could be caused for the diamagnetic behavior of lipase interfering with the superparamagnetism of NPs [60]. Nonetheless, the recovered magnetic moment of the obtained NPs will be sufficiently high to be easily attracted by a 0.1 T permanent magnet. Also SAR values suffered reduction, especially in the case of lipase immobilized on OA-NPs. 

However, in all cases, an increment of temperature of 1, 0.9 and 0.8 °C/min by applying an alternating magnetic field (282 kHz, 20 mT and 1 mg/mL sample concentration) was achieved for CALB_EDA_@CA-NPs, CALB_EDA_@SA-NPs and CALB_EDA_@OA-NPs respectively. It is worth of note that the applied parameters for SAR measurements were the same as used to induce magnetic hyperthermia for human application in clinical practice. It means that these conditions will be easily tolerated also by the immobilized biocatalyst during the magnetic hyperthermia heating. 

### 3.3. Thermal Stability Studies

**Figure 5:**
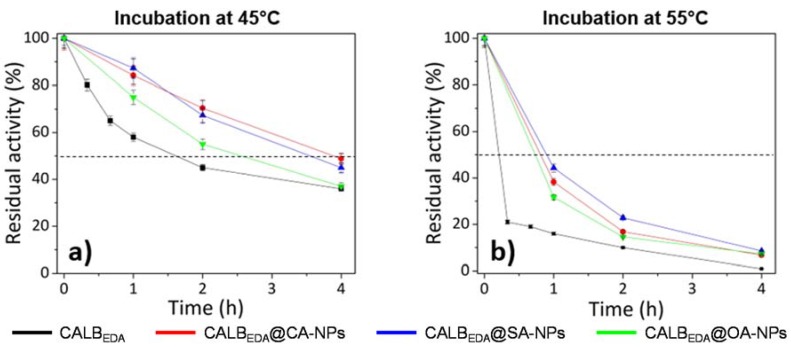
Thermal stability of free and immobilized CALB_EDA_ at different temperatures: (**a**) 45 °C and (**b**) 55 °C.

Independently of the selected heating strategy, one of the goals of this research was focused on the assessment of the impact of immobilization protocol on nanobiocatalyst thermal stability, especially in order to evaluate its potential applicability in high temperature bioprocesses. With this aim, all the three lipases immobilized on magnetic IONPs, together with the free CALB_EDA_ lipase as control, were incubated at 45 and 55 °C during different times and the retained activity was evaluated by hydrolysis of pNPB (Figure 5). At 45 °C, thermal stability of both CALB_EDA_@CA-NPs and CALB_EDA_@SA-NPs covalent derivatives was quite similar, being about 4 h the half-life time (Figure 5a). The CALB_EDA_@OA-NPs was less stable, reaching the half-life after 2.5 h (Figure 5a). However, all immobilized catalysts were revealed to be more thermostable than free CALB_EDA_ since the half-life of this last was reached after 1.5 h (Figure 5a). During incubation at 55 °C, the general trend above described was respected but with faster kinetic of thermal inactivation. In fact, all the nanoderivatives reached half-life values within 45 min and 1 h whereas the free enzyme reached that value only after 10 min incubation (Figure 5b). This general trend of thermal stability results (immobilized enzyme more stable than free enzyme and covalent immobilization catalysts more stable than physical adsorption) was in agreement with similar results reported in literature [61]. 

### 3.4. Kinetic Resolution of Racemic Methyl Mandelate *(**1**)* Using Enzymatic Nanoderivatives

**Scheme 1:**
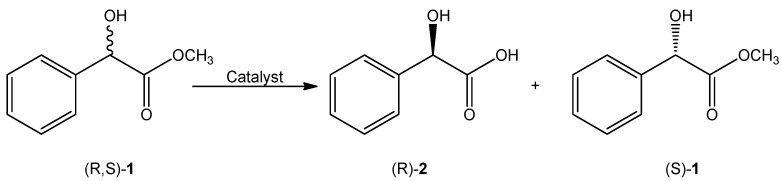
Enzymatic kinetic resolution of *rac*-methyl mandelate (**1**).

The impact of the different immobilization strategies on the catalytic properties of CALB_EDA_ was evaluated by means of the kinetic resolution of racemic methyl mandelate (R,S)-**1** in order to produce the target (*R*)-(−)-mandelic acid ((*R*)-**2**), an important chiral building block widely used in pharmacy and the production of fine chemicals (Scheme 1) [36]. The results of this screening are reported in Table 2. 

Considering the thermal stability study and the potential practical applications, all the catalysts have been compared at 25 °C as well as at 45 °C. The enantioselectivity (*E*) was calculated according to Chen’s method [62] from the reaction conversion and the enantiomeric excess of the substrate (ee_s_). The extent of conversion was estimated by reversed phase HPLC and ee_s_ by chiral HPLC using the individual commercial enantiomers of methyl mandelate as analytical references. In all cases, the (*R*)-ester was preferentially hydrolyzed. As general results, the CALB_EDA_ lipase showed a good specific activity toward this substrate at both assessed temperatures (Table 2). Depending on the immobilization protocol (covalent immobilization vs. hydrophobic adsorption), some crucial differences can be appreciated. At room temperature, the lipase physically adsorbed on the oleic acid hydrophobic layer showed greater activity if compared with both covalent immobilizations (about 1.4 fold, Table 2, entry 3 vs. entries 1 and 2). Analyzing the stereoselectivity properties, all the three derivatives showed good and quite similar results, being the CALB_EDA_@SA-NPs the best catalyst (87% ee, Table 2, entry 2). However, both covalent immobilization of lipase on carboxy surfaces (CALB_EDA_@CA-NPs and CALB_EDA_ @SA-NPs) showed 3 folds higher enantioselectivity value in comparison with lipase physically adsorbed on hydrophobic nanomaterial (CALB_EDA_@OA-NPs), being these values 27, 28 and 9, respectively (Table 2, entries 1–3). When carried out at 45 °C, the kinetic resolution of racemic **1** catalyzed by the same nanobiocatalysts showed the same general trend above described but with better values. In fact, the reaction rates of all the derivatives were notably increased retrieving an overall specific activity increase of almost 3 folds. Also in this case, CALB_EDA_@OA-NPs derivative was confirmed to be the most active catalyst showing 120 IU/mg_lip_ (Table 2, entry 6). The stereoselectivity was also increased even if not following the same proportion. In fact, all the three nanoderivatives showed a quite high and similar enantiomeric excess being newly the CALB_EDA_@SA-NPs the most selective catalyst (almost 90% ee, Table 2, entry 5). The enantioselectivity was increased in all cases, being generally confirmed the covalent immobilization as the best enantioselective strategy and specifically the immobilization of CALB_EDA_ on SA-NPs as the most enantioselective protocol (*E* = 38, Table 2, entry 5).

The recyclability properties of the three nanoderivatives were also evaluated during three different cycles of kinetic resolution of racemic **1** at 45 °C. In these conditions, the covalent nanobiocatalysts retained more than the 90% of their initial activity whereas the physically adsorbed one losses more than 20% of its initial specific activity. In all cases, the enantioselectivity was maintained (Table S1).

### 3.5. Nanocatalyst Storage Studies

One of the most important characteristic to be evaluated in a heterogeneous biocatalyst regards its ability to maintain the original catalytic properties over long storage time (shelf-life). In more details, it is a crucial requirement that the stored biocatalysts follow maintaining their original activity and colloidal properties as long as they are stored.

One of the storage strategies that are being commonly applied in pharmaceutical industry is the freeze-drying. This process is widely used to improve the shelf-life of many pharmaceutical products. However, with this technological process, NPs suspensions can undergo to colloidal destabilization due to the stress generated by freezing and dehydration [63,64].

As results, ferrofluid suspensions can generate great and irreversible aggregates. Furthermore, it has been extensively described as also large biomolecules (e.g., enzymes and proteins) can suffer the conditions required during this process, especially the freezing and dehydration steps [65]. As a consequence, these biomolecules could lose their tridimensional structure finally resulting in a biological activity drop. For all these reasons, cryoprotectants—special excipients able to protect the NPs suspension during freezing—should be added. 

**Figure 6:**
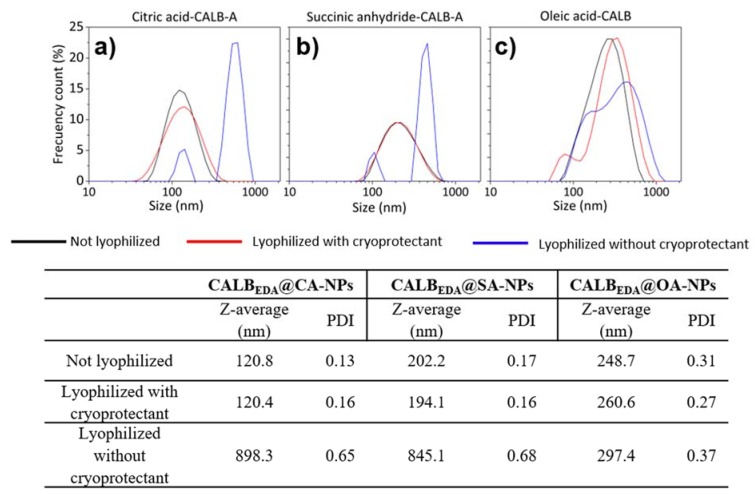
Colloidal properties CALB_EDA_-NPs in suspension and after lyophilization. Hydrodynamic sizes of CALB-immobilized NPs after storage in two different forms: aqueous suspension and freeze-dried. Lyophilized samples, with or without cryoprotectant (red and blue lines) and stored at 4 °C, were re-suspended in phosphate buffer 10 mM before the experiment. (**a**) Hydrodynamic size of CALB_EDA_@CA-NPs, (**b**) hydrodynamic size of CALB_EDA_@SA-NPs and (**c**) hydrodynamic size of CALB_EDA_@OA-NPs.

To study the impact of freeze-drying on nanocatalyst activity and stability, the prepared samples were frozen with and without sucrose (5% *w*/*v*), a well-known cryoprotectant [53]. Colloidal properties were evaluated by measuring the hydrodynamic size of lipase-immobilized NPs (Figure 6). It was observed that the magnetic supports lyophilized in the presence of sucrose showed hydrodynamic sizes and polydispersion index (PDI) values quite similar to the original aqueous suspension of each immobilization. Also hydrodynamic distributions did not change during freeze-drying. When freeze-dried in absence of sucrose, CALB_EDA_@CA-NPs and CALB_EDA_@SA-NPs samples greatly increased their hydrodynamic size, from 120.8 to 898.3 nm and from 202.2 to 845.1 nm respectively. Furthermore, also the PDI increased up to 0.65 as well as even their hydrodynamic distributions. In fact, two differentiated aggregate populations have appeared (Figure 6a,b).

Interestingly, the CALB_EDA_@OA-NPs nanoderivative only underwent to a slight hydrodynamic size increase in presence (from 248.7 up to 260.6 nm) as well as in absence (from 248.7 up to 297.4 nm) of the crioprotectant (Figure 6c). The same trend was observed for PDI values (from 0.31 to 0.37) (Figure 6c). As result, the lipase physically adsorbed on oleic acid coating layer kept a similar aggregation states, independently of the presence of the cryoprotectant. This behavior is probably due to the presence of the fatty acid layer that is able to act as cryoprotectant by itself during the freeze-drying process. 

**Figure 7:**
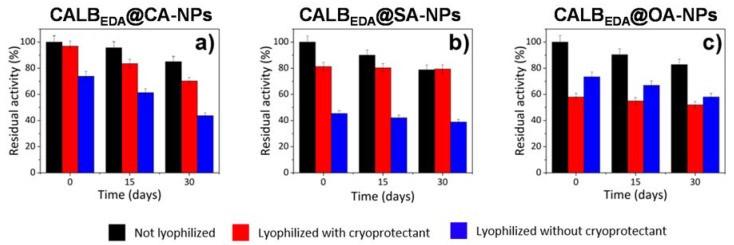
Retained activity of different CALB_EDA_ nanoderivatives stored in aqueous suspension and after lyophilization. Lyophilized samples (stored at 4 °C) were re-suspended in phosphate buffer 10 mM prior to measure the catalytic activity retained by CALB_EDA_ over the storage time. (**a**) Retained activity of CALB_EDA_@CA-NPs, (**b**) retained activity of CALB_EDA_@SA-NPs and (**c**) retained activity of CALB_EDA_@OA-NPs.

The catalyst storage shelf-life was also evaluated in terms of retained activity during 30 days of storage at 4 °C (Figure 7). The three biocatalyst-magnetic nanocarriers complexes were kept in powder and in aqueous solution form for comparison. In the latter case, 80% activity retention was maintained even after 30 days for all the nanocatalysts (Figure 7a–c). 

The presence of the cryoprotectant during freeze-drying produced an initial activity decrease of the enzyme in a different extent depending on the immobilization protocol applied. For example, the CALB_EDA_@OA-NPs hydrophobic derivative underwent to the greatest activity decrease (−40% loss) (Figure 7c) whereas the CALB_EDA_@CA-NPs and CALB_EDA_@SA-NPs covalent derivatives only loss their initial activity in a 5% (Figure 7a) and 20% (Figure 7b) values respectively. In all cases, this initial activity drop was roughly maintained during one month of storage (Figure 7). 

The freeze-drying in absence of cryoprotectant caused a more drastic initial activity drop, especially in the case of covalent nanoderivatives (up to 75% and 45% of recovered activity for CALB_EDA_@CA-NPs and CALB_EDA_@SA-NPs respectively; Figure 7a,b). This result indicates that the initial activity reduction is not only due to freeze-drying process in absence of sucrose but it is also related to the aggregation phenomenon of nanomaterials. This colloidal property issue also generates substrate diffusional problems that negatively impact on the final biocatalyst activity and that are more evident in the case of more hydrophilic nanomaterials (CA-NPs and SA-NPs). This trend is also confirmed by the evidence that hydrophobic CALB_EDA_@OA-NPs nanoderivative better preserved its initial activity in absence of sucrose rather than in its presence (74% vs. 58% of recovered activity; Figure 7c). It is worth of reminding as the sucrose generates a very hydrophilic microenvironment surrounding each lipase molecule and this behavior could negatively impact on the interfacial activation mechanism and the diffusion of hydrophobic substrates. Consequently, the recovered specific activity of lipase immobilized on hydrophobic surface freeze-dried in presence of sucrose is expected to be lower, in agreement with the achieved experimental results. After 30 days storage, CALB_EDA_@CA-NPs has lost an additional 30% in activity, CALB_EDA_@SA-NPs 5% and CALB_EDA_@OA-NPs 16% (Figure 7).

As final general consideration, the freeze-drying process in presence of sucrose as cryoprotectant is the best choice for long term storage of CALB_EDA_ immobilized by covalent attachment on carboxylic coated magnetic nanoparticle and more in details for CALB_EDA_@CA-NPs.

## 4. Conclusions

In sum, we have demonstrated that during the controlled oriented immobilization of lipase B from *Candida antarctica* on magnetic IONPs, the combination between the careful tailoring of nanocarrier surface and the site-specific chemical modification of biocatalyst is a crucial parameter to finely modulate its catalytic behavior. In more details, the lipase was chemically aminated and covalently immobilized on magnetic nanocarrier with polycarboxy surface obtained by previous coating with citric acid (CALB_EDA_@CA-NPs) or succinic anhydride (CALB_EDA_@SA-NPs). By means of an oriented hydrophobic adsorption of the same aminated lipase on oleic acid-coated magnetic IONPs, the CALB_EDA_@OA-NPs nanocatalyst was also achieved. In order to evaluate the overall impact of each immobilization protocol, the physical, chemical and magnetic properties of the as prepared magnetic nanoderivatives have been extensively characterized. With the same scope, the catalytic properties of each CALB_EDA_ nanoderivative (especially in terms of specific activity, enantioselectivity and recyclability) have been compared by carrying out the kinetic resolution of racemic methyl mandelate. Furthermore, the thermal stability has been also investigated. Finally, a freeze-drying process in presence of cryoprotectant has been developed in order to grant good catalytic properties retention over long-time storage (1 month). Taken together, these results underline as each immobilization protocol (considered as the sum of nanocarrier surface engineering *plus* enzyme modification) is able to modulate specifically one property of a selected lipase. Therefore, this combining strategy is here demonstrated to be a powerful tool in the design and preparation of nanobiocatalyst libraries with enhanced properties and broad application scope.

## Figures and Tables

**Table 1:** Physical, chemical and magnetic characterization of citric acid (CA), succinic anhydride (SA) and oleic acid (OA) coated nanoparticles (NPs) with or without aminated CALB immobilized on their surfaces.

Sample	Immobilization		TEM	DLS		ζ-potential	SAR ^a^	Ms
-	µg Enzyme/mg Fe	Enzyme activity (IU ^b^/mg_lip_)	Core size (nm)	Ø_Hydr._Z-average (nm)	PDI ^c^	pH 7 (mV)	W/g_Fe_	emu/g _γ-Fe2O3_
NP	-	-	12.9 ± 2.6	95.5	0.2	+3.45	131	67.9 ± 0.03
CA-NP	-	-	11.7 3.3	80.72	0.19	−25.7	161.2	53.9 ± 0.01
CALB_EDA_@ CA-NP	234	5.73	10.3 ± 4.3	120.8	0.13	−6.2	75.36	31.13 ± 0.02
SA-NP	-	-	13 ± 2.7	121.5	0.15	−13.9	125.6	57.67 ± 0.02
CALB_EDA_@ SA-NP	149	8.28	12.4 ± 2.2	202.2	0.17	−6.2	60.7	36.35 ± 0.01
OA-NP ^d^	-	-	12 ± 2.8	109.2	0.15	-	106.45	64.78 ± 0.04
CALB_EDA_@ OA-NP	162.7	13.5	11.8 ± 2.2	248.7	0.31	−5.7	58.61	49.6 ± 0.03

^a^ Specific absorption ratio (SAR) measurement were made in an alternating magnetic field of 20 mT and 282 kHz. ^b^ International Unit (IU) corresponds to the amount of enzyme which sets free 1 μmol p-nytrophenol per minute at pH 7.0 and 25 °C. ^c^ Polydispersion Index. ^d^ All the measurements were carried out using toluene as solvent.

**Table 2:** Enzymatic kinetic resolution of *rac*-methyl mandelate (**1**) catalyzed by CALB_EDA_ immobilized on different nanocarriers.

Entry	*T* (°C)	Catalyst ^a^	Specific activity (IU/mg_lip_) ^b^	*c* (%)	ee (%) ^c^	*E* ^d^
1	25	CALB_EDA_@CA-NPs	32	48 ^e^	77.6 (R)	27
2	25	CALB_EDA_@SA-NPs	34.6	51.5 ^e^	87 (R)	28
3	25	CALB_EDA_@OA-NPs	46.6	55 ^e^	74 (R)	9
4	45	CALB_EDA_@CA-NPs	83.2	49.5 ^f^	84 (R)	34
5	45	CALB_EDA_@SA-NPs	88	51 ^f^	89 (R)	38
6	45	CALB_EDA_@OA-NPs	120	53 ^f^	80 (R)	14

Hydrolysis of 2 mM methyl dl-mandelate (R,S-**1**) in MeCN-10 mM phosphate buffer, pH 7 (5:95). ^a^ 0.5 mg lip for each reaction. ^b^ The specific activity is expressed as IU/mg of lipase immobilized on each support. IU = μmol of substrate hydrolyzed × minute × amount of lipase. ^c^ Percentage of enantiomeric excess and absolute configuration of the acid **2** were determined by chiral HPLC, using commercial reference samples. ^d^ *E* (enantioselectivity) = ln[(1 − *c*)(1 − ee_s_)]/ln[(1 − *c*)(1 + ee_s_)]; *c*, conversion [62]. ^e^ Conversion calculated by RP-HPLC after 50–60 min. ^f^ Conversion calculated by RP-HPLC after 15–25 min.

## Supplementary Materials

The following are available online at http://www.mdpi.com/2073-4360/10/6/615/s1, Figure S1: TEM micrographs of (a) pristine nanoparticles, (b) CA-coated NPs. (c) SA-coated NPs. (d) OA-coated NPs. (e) CALB_EDA_@CA-NPs. (f) CALB_EDA_@SA-NPs. (g) CALB_EDA_@OA-NPs, Figure S2: FTIR spectra of citric acid and succinic anhydride-coated NP compared to free citric acid and succinic anhydride molecules, Table S1. Recyclability assessment of enzyme nanoderivatives.
