# Supplementary material for: Modulation of the Catalytic Properties of Lipase B from Candida antarctica by Immobilization on Tailor-Made Magnetic Iron Oxide Nanoparticles: The Key Role of Nanocarrier Surface Engineering
Source: Polymers (Basel). 2018 Jun 5;10(6):615. doi: 10.3390/polym10060615 (PMC6404122; doi:10.3390/polym10060615)
Supplement: Supplementary file 1 [file polymers-10-00615-s001.pdf]

Supplementary Materials

Modulation Of The Catalytic Properties Of Lipase B From *Candida Antarctica* By Immobilization On Tailor-Made Magnetic Iron Oxide Nanoparticles: The Key Role Of Nanocarrier Surface Engineering.

Mario Viñambres,[1] Marco Filice,[2,3,4] Marzia Marciello[1*]*

[1] Department of Biomaterials and Bioinspired Material, Materials Science Institute of Madrid (ICMM-CSIC), Sor Juana Inés de la Cruz 3, Cantoblanco, Madrid, Spain

[2] Department of Chemistry in Pharmaceutical Sciences, Faculty of Pharmacy, Complutense University (UCM), Plaza Ramón y Cajal, 28040 Madrid (Spain)

[3] National Research Centre for Cardiovascular Disease (CNIC), C/Melchor Fernández-Almagro 3. 28029, Madrid, Spain.

[4] Biomedical Research Networking Center for Respiratory Diseases (CIBERES), C/Melchor Fernández-Almagro 3. 28029, Madrid, Spain.

[*]Correspondence to: mfilice@ucm.es (MF); marziamarciello@gmail.com (MM)

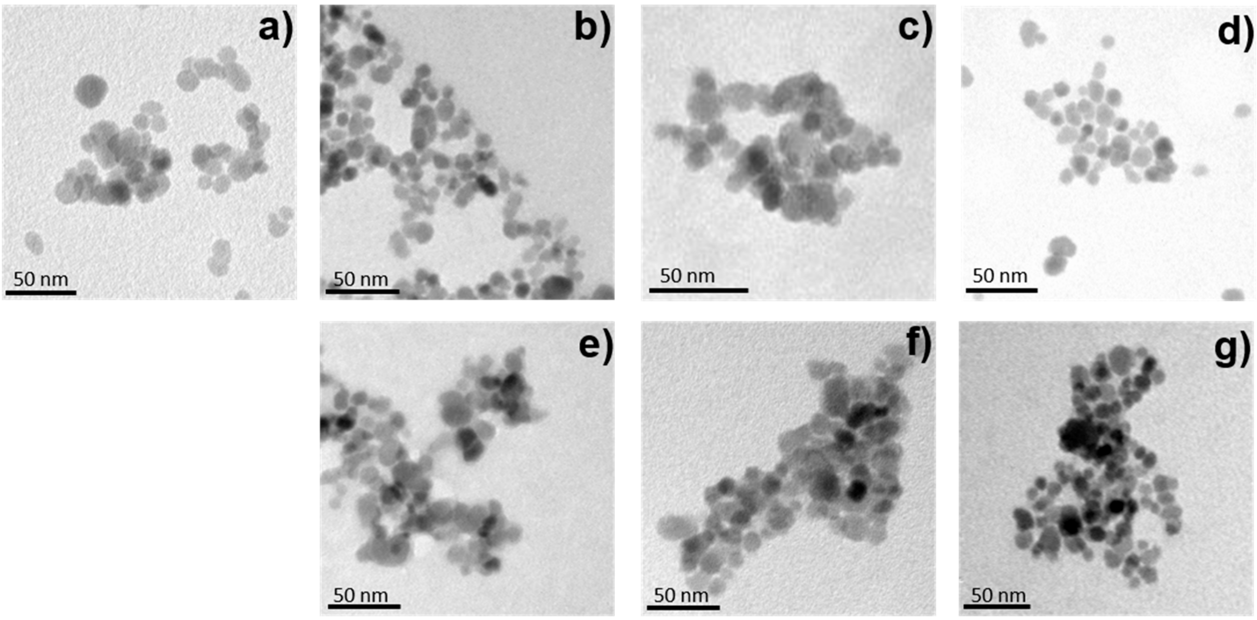

Figure S1: TEM micrographs of a) pristine nanoparticles, b) CA-coated NPs. c) SA-coated NPs. d) OA-coated NPs. e) $CALB_{EDA}$@CA-NPs. f) $CALB_{EDA}$@SA-NPs. g) $CALB_{EDA}$@OA-NPs.

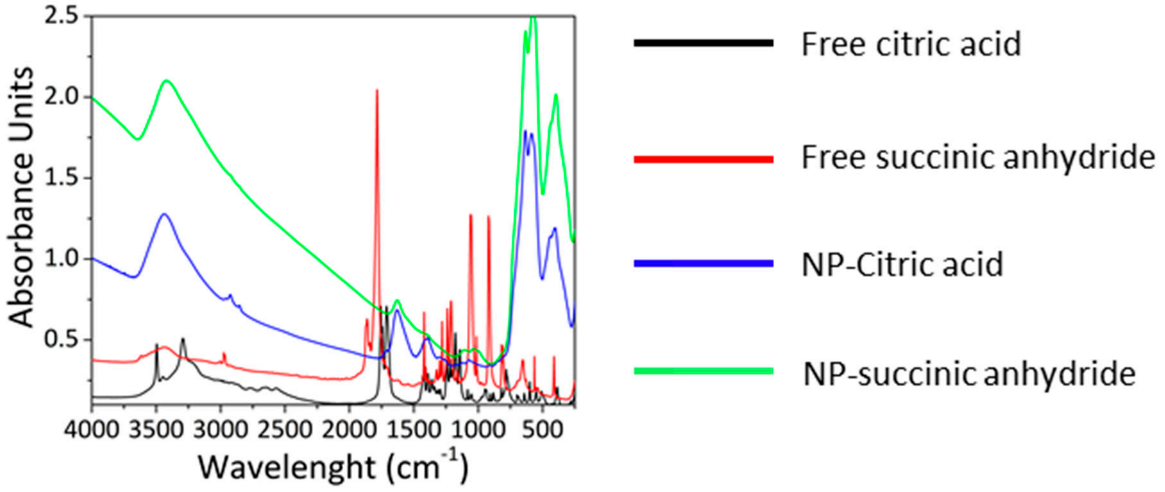

Figure S2: FTIR spectra of citric acid and succinic anhydride-coated NP compared to free citric acid and succinic anhydride molecules.

FTIR spectra of hydrophilic coated-NPs compared with the respective free molecules (citric acid and succinic anhydride) are reported in Figure S2. The peaks at around 1800-1700 cm^{-1} in the free molecules, corresponding to asymmetric and symmetric COO- stretching, have been shifted to the right when conjugated to the NPs. The intensity of the peaks between 1500-750 cm^{-1} was attenuated in the case of coated NPs due to the high amount of iron oxide nanoparticles respect to the mass of coating in the sample.

Table S1. Recyclability assessment of enzyme nanoderivatives.[a]

	$CALB_{EDA}$@CA-NPs		$CALB_{EDA}$@SA-NPs		$CALB_{EDA}$@OA-NPs	
**Cycle**	**Specific Activity (IU/mg_{lip})**[b]	***E***	**Specific Activity (IU/mg_{lip})**[b]	***E***	**Specific Activity (IU/mg_{lip})**[b]	***E***
1	32	27	34.6	28	46.6	9
2	30	26.5	32.4	27.5	40	9
3	29.5	26.7	31	26.5	37	8

[a] Hydrolysis of 2 mM methyl DL-mandelate (R,S-**1**) in MeCN-10 mM phosphate buffer, pH 7 (5:95) at 45°C.

[b] The specific activity is expressed as IU/mg of lipase immobilized on each support. IU = µmol of substrate hydrolyzed x minute x amount of lipase.
